# A coral-derived neuropeptide suppresses pentylenetetrazol (PTZ)-induced epileptic seizures and improves recognition memory deficits by modulating NPY-Y1R

**DOI:** 10.1007/s00204-025-04164-3

**Published:** 2025-09-26

**Authors:** Qian Chen, Congshuang Deng, Xiaoshan Huang, Aili Wang, Nan Xu, Kaixun Cao, Min Yang, Shang Li, Qiumin Lu, Guiyi Gong, Simon Ming-Yuen Lee

**Affiliations:** 1 Center for Evolution and Conservation Biology, Southern Marine Science and Engineering Guangdong Laboratory (Guangzhou), Guangzhou, 511458 China; 2 Shenzhen Academy of Environmental Sciences, Shenzhen, 518022 Guangdong China; 3 Engineering Laboratory of Peptides of Chinese Academy of Sciences, Key Laboratory of Bioactive Peptides of Yunnan Province, KIZ-CUHK Joint Laboratory of Bioresources and Molecular Research in Common Diseases, National Resource Center for Non-Human Primates, National Research Facility for Phenotypic & Genetic Analysis of Model Animals (Primate Facility), State Key Laboratory of Genetic Evolution & Animal Models, Sino-African Joint Research Center, New Cornerstone Science Laboratory, Kunming Institute of Zoology, The Chinese Academy of Sciences, No.17 Longxin Road, Kunming, 650201 Yunnan China; 4 State Key Laboratory of Quality Research in Chinese Medicine, Institute of Chinese Medical Sciences, University of Macau, Macao, 999078 China; 5 College of Life Sciences, Nanjing Agricultural University, Nanjing, 210095 Jiangsu China; 6 Laboratory for Accelerated Vascular Research, Department of Surgery, University of California San Francisco, San Francisco, CA 94143 USA; 7 Department of Food Science and Nutrition, The Hong Kong Polytechnic University, Hung Hom, Hong Kong, 999077 China; 8 PolyU-BGI Joint Research Centre for Genomics and Synthetic Biology in Global Ocean Resources, Hung Hom, Hong Kong, 999077 China

**Keywords:** Coral, Neuropeptide Y receptor, Epilepsy, Oxidative stress, Inflammation, Excitotoxicity

## Abstract

Epilepsy is a prevalent neurological disorder characterized by recurrent and unprovoked seizures. Despite the availability of anti-epileptic drugs (AEDs), a significant number of patients are still suffering from drug-resistant epilepsy. Neuropeptide Y (NPY) signaling system has emerged as a potential target for the development of anti-epileptic drugs due to its modulation of epileptic activity. In this study, we investigated the therapeutic potential of our previously discovered Scleractinia-derived NPY-like peptide (TpNPY) in seizure disorders. The anticonvulsant effects of TpNPY were evaluated using PTZ-induced seizures in zebrafish and mice in vivo. Furthermore, the underlying molecular mechanisms of TpNPY were assessed using glutamate-induced excitotoxicity models in HT22 mouse hippocampal cells in vitro. Our findings indicated that TpNPY could alleviate PTZ-induced seizure behavior, reduce the expression of seizure-associated immediate-early genes and the production of Reactive Oxygen Species (ROS) in zebrafish. In mice, TpNPY improved seizure behaviors, decreased inflammatory cytokine levels, and ameliorated abnormal glial activation in a PTZ kindling epileptic model. Besides, the administration of TpNPY could attenuate the PTZ-induced anxiety levels and improve recognition memory deficits. Moreover, TpNPY promotes neurogenesis and neural synaptic plasticity through the BDNF/TrkB signaling pathway. Additionally, TpNPY restored cell injury and attenuated oxidative stress in glutamate-challenged HT22 cells through the Nrf2/HO-1 signaling pathway. These results highlight the potential therapeutic efficacy of TpNPY in the treatment of seizures and provide new insights into the development of coral-derived anti-epileptic peptide-based drugs.

## Introduction

Epilepsy is a prevalent, chronic and severe neurological disorder that affects approximately 70 million individuals globally (Thijs et al. 2019). It is characterized by the occurrence of repetitive, spontaneous seizures that result from abnormal and synchronized electrical discharges in the patients’ brain (Amin et al. 2022). Clinically, seizures manifest as transient disturbances in consciousness, motor control (e.g., convulsions), and sensory/motor function (Baxendale et al. 2012). The etiopathogenesis of epilepsy involves complex interactions between genetic predispositions (e.g., ion channel mutations) and acquired insults (e.g., traumatic brain injury, infections) (Velisek et al. 2013). Despite the widespread use of anti-epileptic drugs (AEDs), approximately 30% of patients develop pharmaco-resistant epilepsy, underscoring the limitations of current therapies that prioritize symptomatic seizure suppression over disease-modifying mechanisms (Kwan et al. 2011). This is due in part to the primary focus of most clinical AEDs on alleviating seizure syndromes rather than targeting the potential mechanisms of epilepsy (Velisek et al. 2013). In addition, currently prescribed AEDs exhibit significant adverse effects on epileptic patients, including cognitive impairment and metabolic disturbances (Cramer et al. 2010). Consequently, therapies targeting epileptogenic mechanisms rather than seizure symptoms are urgently needed to improve clinical outcomes.

The hippocampus, a critical brain region situated in the medial temporal lobe, exerts a substantial influence on the pathogenesis and manifestation of epilepsy (Madden and Sutula 2009). Within this region, various types of neurons, including pyramidal cells and inhibitory interneurons, contribute to its overall function (Whitebirch et al. 2022). Under certain conditions, such as excessive stimulation of excitatory neurotransmitters or trauma, pyramidal cells may become excessively excitatory, while inhibitory interneurons may fail to effectively regulate this activity. This imbalance induces hippocampal hyperexcitability, a key factor in seizure initiation (Devinsky et al. 2013). Glutamate-mediated excitotoxicity has been proposed as one key mechanism contributing to the progression and development of epilepsy (Hynd et al. 2004). Briefly, abnormally increased levels of glutamate trigger a large influx of calcium ions into the neurons and prevent the synthesis of intracellular glutathione through cystine/glutamate antiporter inhibition, consequently resulting in the excessive accumulation of reactive oxygen species (ROS) and finally leading to neuronal apoptosis (Savolainen et al. 1998). Meanwhile, accumulating clinical and experimental evidence demonstrates the involvement of brain inflammation in the pathophysiology of human epilepsy (Vezzani et al. 2011). It is reported that pro-inflammatory cytokines from the brains of epileptic patients, including interleukin (IL)−6, IL-1β, and tumor necrosis factor (TNF)-α, are verified to be upregulated during seizure attacks (Gao et al. 2018). Furthermore, epileptic seizures intensify neuroinflammation by affecting signaling pathways associated with COX-2, TGF-β, and Toll-like receptors, which in turn causes detrimental synaptic changes and neuronal hyper-excitability (Murugan et al. 2024).

Neuropeptide Y, a polypeptide consisting of 36 amino acid residues, belongs to the neuroendocrine peptide family known as NPY (Li et al. 2019). It is abundantly distributed in mammalian bodies, particularly in the central nervous system (Wahlestedt et al. 1989). Since the early 1990 s, NPY has garnered substantial attention as a potent endogenous mediator of epileptic activity (Colmers and El Bahh 2003). This is supported by the fact that both epileptogenic process and epilepsy itself alter the expression levels of NPY and NPY receptor encoding genes (Cattaneo et al. 2020). Additionally, NPY could regulate network excitability and homeostasis through its modulatory effects (Cattaneo et al. 2020). Mounting evidence highlights that the administration of exogenous NPY effectively suppresses various types of epileptiform activity (Baraban 2002; Klapstein and Colmers 1997; Woldbye et al. 1997). Therefore, molecules that modulate the NPY signaling system represent a promising source in the development of antiepileptic drugs.

In our previous investigation, we identified an NPY-like peptide (TpNPY) derived from the scleractinia *Turbinaria peltate* and demonstrated its ability to alleviate lipopolysaccharide (LPS)-induced astrocytic inflammation (Chen et al. 2022b). Given its ability to modulate NPY signaling and neuroinflammation, the therapeutic potential of TpNPY for seizures has attracted our attention. However, its exact targets, efficacy in treating epilepsy, and underlying mechanisms still remain unclear. In this study, the potential anticonvulsant effects of TpNPY were further evaluated using PTZ-induced seizure models in zebrafish and in mice in vivo, respectively. Additionally, since hippocampal cells and glutamate play vital roles in the occurrence of seizures, the molecular mechanisms of TpNPY were investigated using glutamate-induced excitotoxicity models in HT22 mouse hippocampal cells in vitro. Besides, we also evaluated the neuroprotective effects of TpNPY on PTZ-induced recognition memory deficits and neurogenesis, as well as neural synaptic plasticity. We hope that our findings will offer a new insight into the development of lead compounds of coral-derived anti-epileptic peptide-based drugs.

## Materials and methods

### Solid phase chemistry synthesis of TpNPY

TpNPY was synthesized via solid-phase peptide synthesis (Hangzhou Go Top Peptide Biotech Co., Ltd, China), which was in accordance with our previous study (Chen et al. 2022b). The synthesis was initiated using Fmoc-Ile-Wang resin with a loading capacity of 0.40 mmol/g, which was first swelled and subsequently subjected to Fmoc deprotection. Sequential coupling reactions were performed from the C-terminus to the N-terminus in accordance with the target peptide sequence. All amino acids were protected at the α-amino group with Fmoc, and the completion of each coupling step was confirmed by a negative indole-3-ketone test. Following the completion of the coupling reactions, the peptide was cleaved from the resin, and side chain protecting groups were removed using a cleavage solution composed of trifluoroacetic acid (TFA), triisopropylsilane, and water (95:2.5:2.5, v/v/v). The crude peptide was dissolved in a water-acetonitrile mixture and subjected to semi-preparative chromatography for initial purification. The purity of the peptide (> 95%) was verified by analytical reversed-phase high-performance liquid chromatography (RP-HPLC) and mass spectrometry (MS). Deprotection and cleavage were achieved using trifluoroacetic acid in water, followed by precipitation of the crude peptide with chilled diethyl ether. The final peptide product was further purified by preparative HPLC, lyophilized, and stored at −20 °C for subsequent studies.

### Structure modeling and molecular dynamics simulation

The three-dimensional structure of the TpNPY peptide was predicted using the AlphaFold2 modeling system and the relaxed_ranked_001 structure was selected (Jumper et al. 2021). The modeled structure of TpNPY was placed within a box containing TIP3P solvent and 150 mM NaCl ions using the GROMACS 2020 software and CHARMM 36 force field (Lee et al. 2016). Then, the system underwent energy minimization, NVT equilibration, NP equilibration, and a 50 ns molecular dynamics simulation.

### Surface plasmon resonance (SPR) analysis

Surface plasmon resonance (SPR) analysis was performed according to the manufacturer’s protocol. Briefly, a CM5 sensor chip (29,149,603, Cytiva, USA) was activated by injecting a mixture of 0.4 M 1-ethyl-3-(3-dimethylaminopropyl) carbodiimide hydrochloride (EDC) and 10 mM N-hydroxysuccinimide (NHS) at a flow rate of 5 μL/min for 20 min. The NPY-Y1 receptor (NPY-Y1R; ZY999Hu013, HZbscience, USA) was diluted to a concentration of 20 μg/mL in 10 mM sodium acetate buffer (pH 5.0) and immobilized onto the activated chip surface until the desired response unit (RU) value was achieved. Residual activated sites on the chip were subsequently blocked by injecting 45 μL of 1 M ethanolamine (pH 8.5). Real-time binding interactions were monitored using a Biacore S200 instrument (USA) at a flow rate of 30 μL/min. To determine the equilibrium dissociation constant (KD) of the interaction between NPY-Y1R and TpNPY, a series of TpNPY dilutions (25, 12.5, 6.25, 3.125, 1.56, and 0.78 μM) prepared in 150 mM phosphate-buffered saline (PBS, pH 7.4) were injected over the NPY-Y1R-immobilized chip surface. Binding kinetics were analyzed using the BIA evaluation software to calculate the *KD* value.

### Cell culture and cell viability

The HT22 cell line was obtained from the ATCC (Manassas, VA, USA) and cultured in DMEM medium with an additional 10% fetal bovine serum (Gibco, US origin), 1% penicillin (100 U/mL) and streptomycin 100 (µg/mL) in a humidified 5% CO_2_ atmosphere at 37 °C. Cell viability was evaluated by measuring the activity of mitochondrial dehydrogenases with 3-(4,5-dimethyl-2-thiazolyl) 2,5 diphenyl-2H-tetrazolium bromide (MTT) assay (Sigma-Aldrich, USA). Briefly, HT22 cells were seeded in 96-well plates at a density of 8 × 10^3^ cells per well. After various drug incubation, cells were incubated in 0.5 mg/mL MTT solution for 4 h at 37 °C. The supernatant medium was then discarded, and 100 µL of DMSO per well was added to dissolve the violet-formazan crystals. The absorbance at 570 nm wavelength was then tested by SpectraMax M5 (Wallac, The Netherlands). Under the same cell culture conditions as the cell viability assay, cytotoxicity was also evaluated via LDH release using an LDH assay kit (Beyotime, China). After different treatments, the supernatant of the cell culture medium was collected and tested according to the manufacturer’s protocol. The absorbance at 490 nm wavelength of supernatant was recorded by SpectraMax M5 (Wallac, The Netherlands).

### Live-dead cell staining

HT22 cells were initially seeded in 24-well plates at a density of 5 × 10^4^ cells per well overnight. After different treatments, the old culture medium was then discarded. Next, the cells were washed three times using PBS. Then, 100 μL Calcein AM/PI detection working solution was added to each well, and the plates were further incubated in a Light-protected environment for 30 min. After incubation, the staining effects were observed under a fluorescence microscope.

### Immunofluorescence for Nrf2 translocation

HT22 cells were seeded in 24-well plate till 70% confluence. Following different treatments, the old culture medium was then discarded. After triple wash with PBS, the cells were fixed with 4% paraformaldehyde (PFA) for 20 min. After washing the cells with PBS twice, cells were incubated with 0.1% Triton X-100 on ice for 15 min to permeabilize the cell membranes and then blocked with 2% bovine serum albumin (BSA) at room temperature for 60 min, followed by the primary anti-Nrf2 antibody (Cell Signaling Technology, Inc) incubation at 4 ℃ overnight. Then, a dye-labeled secondary antibody and 5 μL DAPI were added and incubated for 60 min and 10 min in turn. After washing three times by PBST (PBS with 0.1% Tween 20), the immunoreactivity of cells was detected by Leica DMI8 inverted fluorescence microscope (Leica, Germany).

### Measurement of ROS production

HT22 cells were pre-seeded in 24-well plates (5 × 10^4^ cells/well) and cultured overnight. After glutamate incubation in the presence or absence of TpNPY for 12 h, the plates were washed with PBS twice and dyed in the dark with 5 μM fluorescent probe CM-H2DCFDA for 20 min at 37 °C. The fluorescence intensity was then recorded with flow cytometry (BD Accuri ™ C6, USA) and analyzed by using FlowJo software (FlowJo, LLC, USA).

### Measurement of cytosolic calcium concentration

Cells were prepared as described above. After treatment with TpNPY in the presence or absence of glutamate for 12 h, the cell-permeant Fluo-4 AM fluorescence indicator (Invitrogen) was added and incubated for another 30 min, followed by twice PBS washing and resuspending with a final volume of 500 μL. The intracellular calcium levels were measured by flow cytometry. Light was avoided in the whole process.

### Measurement of lipid peroxidation

The GSH, GSSG concentration was determined using a commercial kit according to the manufacturer’s instructions (Jiancheng, Nanjing, China). MDA, which is a product of lipid peroxidation, is usually used as an indicator of lipid peroxidation (Tsikas 2017). In this study, the MDA level was measured using a thiobarbituric acid reactive species (TBARS) assay kit (Jiancheng, Nanjing, China). The SOD activity was tested using a commercial kit (Beyotime, China).

### Western blot

Sample groups were collected and extracted from cells and mice brain tissues, respectively. For protein sample preparation, the extraction samples were centrifuged at 12,500 rpm at 4 ℃. Then, the supernatants were mixed with SDS-PAGE protein loading buffer (Biosharp, China), protein was denatured by heating at 99 ℃ for 10 min, electrophoresis was performed on 10% SDS-PAGE for 1.5 h, followed by transferring the protein from gel to 0.2 μm PVDF membranes at a constant current for 20 min. Membranes were then blocked with 5% BSA (Biofroxx, Germany) in TBST buffer containing 150 mM NaCl, 10 mM Tris, 0.1% Tween-20, PH 7.4 for 1.5 h, and further incubated with target primary antibodies in TBST buffer at 4 ℃ overnight. After three washes with TBST, the blots were incubated with HRP-conjugated secondary antibodies in TBST at room temperature for 1 h. Finally, the membranes were stained using a high-sig ECL Western blotting detection kit (Tanon, China). Tanon 5200 Multi imaging system (China) was used to visualize the protein bands. The density of each protein band was determined using ImageJ software. The primary antibodies used were as follows: anti-Iba-1, anti-GFAP, anti-BDNF, anti-TrkB, anti-Nrf2, anti-HO-1, anti-Lamin B1, anti-GAPDH were obtained from ZEN-BIOSCIENCE company (China).

### Zebrafish maintenance

The zebraish experiments were approved by the Animal Research Ethics Committee, University of Macau (approval no: UMARE-021b-2020). The AB wild-type zebrafish were strictly manipulated according to the Zebrafish Handbook. Briefly, zebrafish were generated by natural pairwise mating within 3–12 months old and reared at 28.5 °C in a thermo-static environment with a 14 h:10 h light/dark cycle. They were fed with brine shrimp twice daily and occasionally with general tropical fish food.

### Evaluation of zebrafish survival and the locomotor behavior

The 6-day post-fertilization (6-dpf) AB wild-type zebrafish were treated with various concentrations of TpNPY for 24 h in zebrafish-specific E3 culture medium. The mortality of zebrafish was calculated based on the observation of their heartbeat. To trigger seizures, zebrafish larvae were subjected to zebrafish culture medium containing 10 mM PTZ (Sigma-Aldrich, purity ≥ 99%) in 96-well plates. Their locomotor behaviors were recorded using a digital video tracking system (ViewPoint Behavior Technology, France). The swimming pattern and distance at high velocity were observed for 1 h and at 10 min intervals, respectively.

### ROS detection in zebrafish

After treatment with different concentrations of TpNPY with or without PTZ for 24 h, zebrafish larvae were incubated with 10 μM fluorescent probe CM-H2DCFA (Sigma-Aldrich) for 1.5 h at 28.5 °C in dark. Excessive CM-H2DCFA was then washed out by zebrafish E3 culture medium. Subsequently, zebrafish larvae were monitored by an inverted fluorescence microscope (Leica M205FA, Germany) and images were captured in FITC mode. Finally, the integrated intensity of ROS was quantified using ImageJ software.

### Total RNA extraction, reverse transcription, and real-time PCR analysis in zebrafish

The 6-dpf AB wild-type zebrafish were treated with indicated concentrations of TpNPY for 24 h. Zebrafish larvae were collected and further incubated with or without 10 mM PTZ for 30 min. The total RNA of zebrafish was obtained by using Trizol reagent and cDNA was synthesized using a commercial reverse transcription assay kit (Thermo HIGH CAP cDNA kit). The qRT-PCR experiment was conducted using a FastStart Universal SYBR Green reagent (Roche, Switzerland) in LightCycler96 Real-Time PCR Systems (Roche, Switzerland). The sequences of the primers used in the qRT-PCR assay are shown in Table 1.

**Table 1:** List of the primers

Primer Name	Forward Primer sequences	Reverse Primer sequences
*c-fos*	5'-TTACCCGCTCAACCAGACTC-3'	5'-TGACAGTTGGCACGAAAGAG-3'
*npas4a*	5'-GAGTAACCTGGTGCCTCCAA-3'	5'-TTTGCCTACGCACTGATTTG-3'
*ef1a*	5'-GCTCAAACATGGGCTGGTTC-3'	5'-AGGGCATCAAGAAGAGTAGTACCG-3'

### Animals

All mice experimental procedures involving animals were conducted in accordance with the guidelines and regulations stipulated by the approval of the Ethics Committee for Laboratory Animal Welfare of the Kunming Institute of Zoology, Chinese Academy of Sciences (approval number: IACUC-RE-2022–12-005). Adult male C57BL/6 J mice weighing 22 ± 2 g (8 weeks old) were procured from Beijing Vital River Laboratory Animal Technology Co., Ltd. (Beijing, China). The mice were housed under standard laboratory conditions, with a constant room temperature (25 ± 2 °C) and humidity (40%−60%). A 12-h light/dark cycle was maintained in the mouse housing room, and the mice were allowed to eat and drink water comfortably.

### PTZ-kindling-induced epileptic mice model

The PTZ-kindling-induced epileptic mice model was monitored as previously described (Singh et al. 2021). PTZ (30 mg/kg, subconvulsive dose) was dissolved in saline and administered via intraperitoneal injection. TpNPY was dissolved in saline and delivered through daily intravenous injection of increasing concentrations (0.25 mg/kg, 0.5 mg/kg and 1 mg/kg) 30 min before PTZ injections for 14 consecutive days. Randomized grouping was used to divide C57BL/6 mice into 7 groups with 10 mice in each group. Then, behavioral observation was carried out at the same time, until the seizure of mice was kindled. The PTZ-only group was not exposed to TpNPY, and the control group was only given saline.

### Open field test (OFT)

The Open field test (OFT) and was used to evaluate the anxiety level in mice (Kraeuter et al. 2019). After different treatments for 14 days, eight-week-old male C57BL/6 J mice were individually introduced into the periphery of a novel open field arena (44 cm × 44 cm × 30 cm) in a dimly lit room (20–30 lx) and allowed to freely explore for 10 min. Behavioral parameters, including time spent, distance moved, and the number of entries into two defined zones, the periphery (5 cm from the walls) and the center (14 cm × 14 cm), were recorded using a digital video camera connected to the ANY-maze Video Tracking System. Between trials, the open field arenas were thoroughly wiped with 70% ethanol and allowed to dry to eliminate residual odors.

### Morris water maze (MWM) test

The Morris Water Maze (MWM) test assessed spatial learning and memory in mice (Bromley-Brits et al. 2011). A circular pool (opaque with milk powder) was divided into four quadrants, with a hidden platform submerged in the third quadrant. Eight-week-old male C57BL/6 J mice were trained over five days (4 trials/day) to locate the platform, guided by visual cues. Escape latency (max 60 s) and platform stay time (30 s) were recorded. A probe trial (platform removed) was conducted 24 h post-training, recording time to the platform site, crossings, speed, and distance via a video camera connected to the ANY-maze Video Tracking System.

### Novel object recognition (NOR)

The novel object recognition (NOR) test was conducted following established protocols (Leger et al. 2013) using a square open-field arena (44 × 44 × 30 cm) containing fixed objects of varying shapes, sizes, and textures. The testing protocol comprised two phases: (1) a 5-min acquisition phase where mice explored two identical objects (12 cm height × 7 cm base diameter) placed in the arena, and (2) a 5-min retrieval phase conducted 2 h later, during which one familiar object was replaced with a novel cubic object (6.5 × 6.5 × 6.5 cm). Exploration behavior (defined as nose contact within ≤ 2 cm or active investigation) was video-recorded for subsequent analysis of time spent with each object type. Between trials, the apparatus was thoroughly cleaned with 75% ethanol. Recognition memory was quantified using a discrimination index calculated as: [time exploring novel object/(time exploring novel + familiar objects)].

### BrdU/NeuN double staining

The mice in different treatment groups were intraperitoneal injection daily with BrdU (50 mg/kg) for 14 days. Mice were then sacrificed after induction, confocal fluorescence analysis of cells double-labeled for bromodeoxyuridinc (BrdU) and NeuN (ab104224, Abcam, UK) in the DG were then performed and photographed.

### Golgi-Cox staining

Mice brain tissue derived from the CA3 region of mice was excised and subsequently stained with Golgi-Cox staining solution at 4 ℃ in the dark for a period of 14 days, with the solution being replaced every 2 days. The brains were serially sectioned into 150-mm-thick coronal sections by employing a vibratome. All slices were immersed in distilled water for 1 min, 70% alcohol for 10 min, 90% alcohol for 15 min, and 100% alcohol for 20 min. Ultimately, the sections were subjected to defatting in a xylene substitute. The slices were mounted onto slides using neutral balsam and cover glasses. Dendritic spines within the CA3 region were photographed morphologically by a light microscope.

### ELISA assay

The TNF-α, IL-6, and IL-1β were assessed and quantified by specific ELISA Ready-SET-Go kits (eBiosciences, San Diego, CA, United States) following the manufacturer’s protocols.

### Statistical analysis

All data are expressed as Means ± SEM from at least three independent experiments. Statistical analysis was performed using GraphPad Prism 7.0 software. Statistical significance was assessed by One-way ANOVA analysis followed by post-hoc Dunnett’s test and post-hoc Tukey’s test, and a *p*-values less than 0.05 was considered statistically significant.

## Results

### TpNPY exhibits direct binding capability to NPY-Y1R, as verified by SPR analysis

**Fig. 1:**
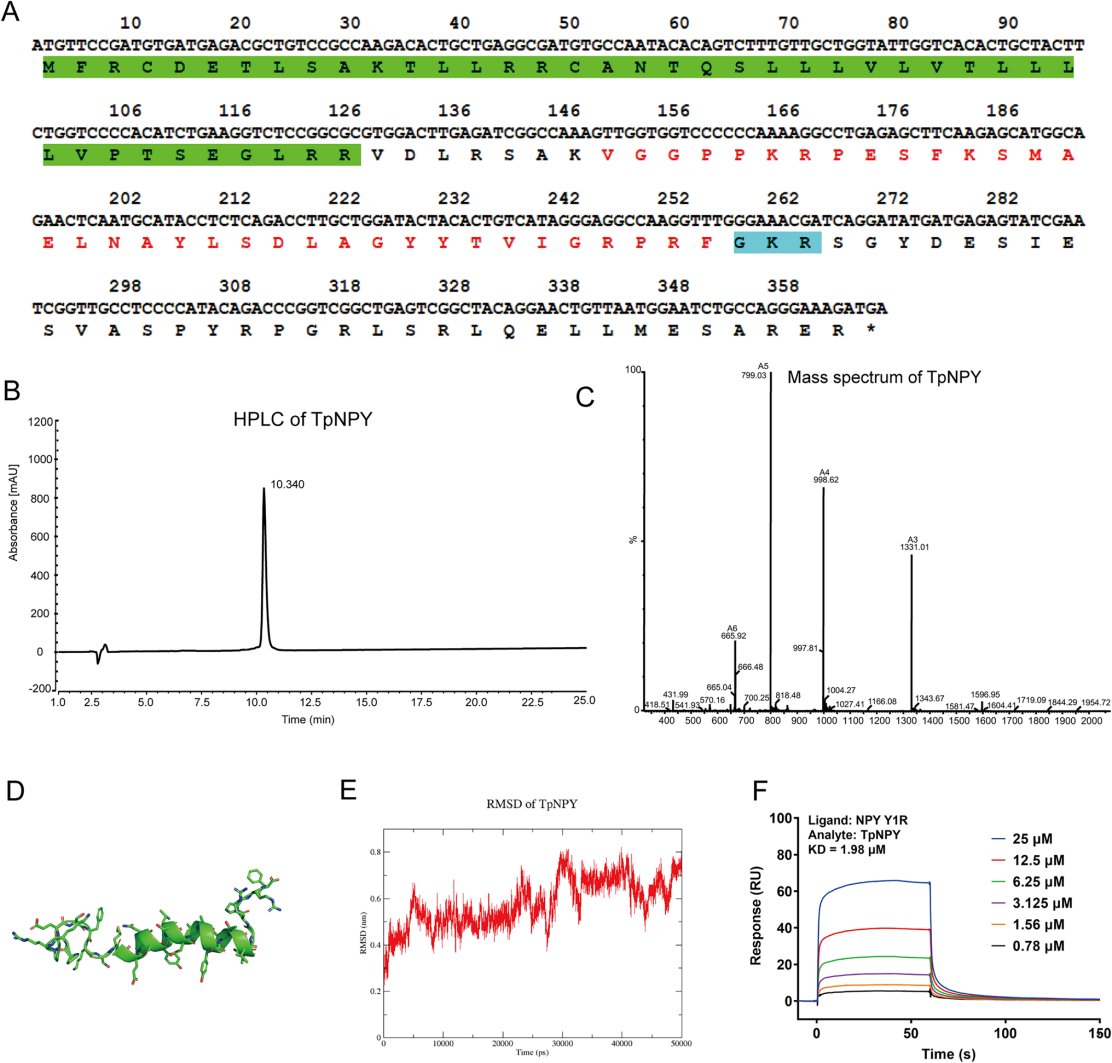
TpNPY can directly interact with NPY-Y1R. **A** The full-length sequences of cDNA and amino acid residues of TpNPY. Putative signal peptides are highlighted in green, NPY-like contig and precursor-related amidated sequences are indicated in red and blue, respectively. **B** Analytical HPLC of TpNPY. **C** Mass spectrum of TpNPY. **D** The three-dimensional structure of TpNPY by structural modeling. **E** The RMSD of TpNPY in 50 ns dynamic simulation. **F** The binding interaction between TpNPY and NPY Y1R was investigated by the SPR technique

In our previous study, we successfully obtained an NPY-like peptide from the transcriptome of *T. peltate* (Chen et al. 2022b). The full-length sequence of this peptide is depicted in Fig. 1A, the mature TpNPY peptide sequence was “VGGPPKRPESFKSMAELNAYLSDLAGYYTVIGRPRF-NH_2_”. The peptide was chemically synthesized for functional investigation (Fig. 1, B, C). Through molecular dynamic simulation, it was demonstrated that the three-dimensional structure of TpNPY (Fig. 1, D) exhibited remarkable stability, with a Root Mean Square Deviation (RMSD) fluctuation below 0.8 nm (Fig. 1E). Our previous findings confirmed that TpNPY interacts with NPY-Y1R via in silico molecular docking and was further validated using specific NPY-Y1R antagonists in cell models (Chen et al. 2022b). As shown in Fig. 1F, the surface plasmon resonance (SPR) analysis revealed a direct interaction between TpNPY and NPY-Y1R, with an equilibrium dissociation constant of 1.98 μM, further confirming their binding affinity.

### TpNPY attenuated the hyperactivity, the upregulation of seizure-associated immediate-early genes and ROS overproduction in PTZ-elicited zebrafish larvae

**Fig. 2:**
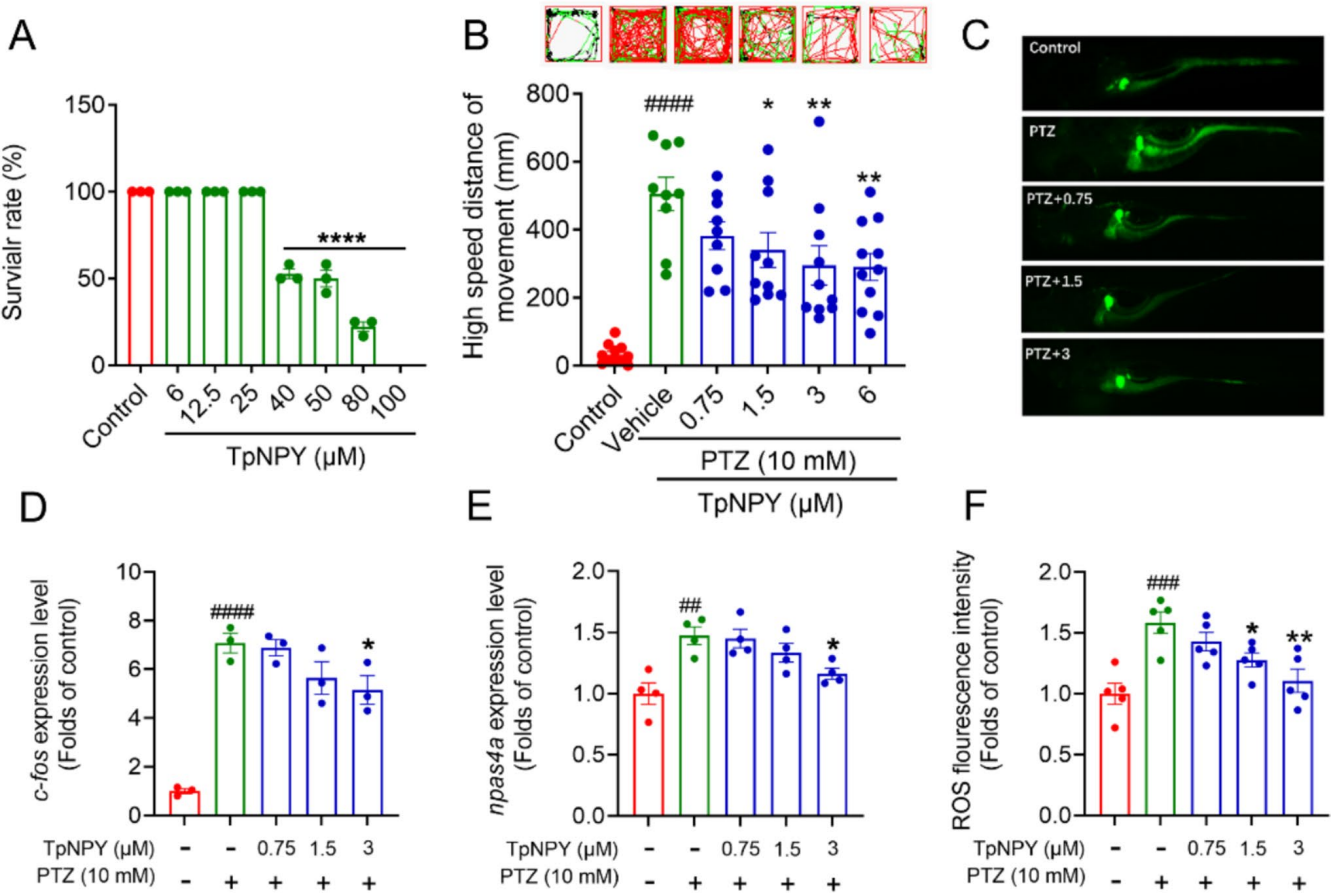
TpNPY suppressed the epileptic locomotor behavior, reversed the upregulation of seizure-associated immediate-early genes and prevented ROS overproduction in zebrafish larvae challenged with PTZ. **A** The mortality of zebrafish larvae after exposure to increasing concentrations of TpNPY for 24 h, ^****^P < 0.0001 vs control group. **B** TpNPY reversed PTZ-triggered alteration of swimming behaviors in zebrafish larvae. **C** The images of ROS fluorescence intensity of zebrafish larvae upon different treatments. **D** and **E** The *c-fos* and *npas4a* expression levels in zebrafish larvae upon different treatments. **F** The quantitative results of ROS fluorescence intensity of zebrafish in different treatment groups. Data are shown as Means ± SEM (*n* ≥ 3). ^##^*P* < 0.01, ^###^*P* < 0.001, ^####^*P* < 0.0001 vs control group, ^*^P < 0.05, ^**^ *P* < 0.01, ^***^P < 0.001 vs PTZ group

Various animal-based epileptic models have been employed to investigate the physiological basis of epilepsy and to facilitate the development of anti-epileptic drugs (Löscher 2017). The PTZ-induced zebrafish epilepsy model offers high efficiency and experimental convenience (Kolesnikova et al. 2022). In this study, the potential anti-epileptic effects of TpNPY were initially assessed using a PTZ-induced seizure model in zebrafish. The results depicted in Fig. 2A indicated that TpNPY at concentrations of 6–25 μM did not lead to any substantial changes in the survival rate of zebrafish larvae. However, once the concentration arrived at 40 μM, the mortality rate of zebrafish larvae sharply increased, reaching approximately 50%. As demonstrated in Fig. 2B, the application of 10 mM PTZ dramatically altered the swimming pattern of the zebrafish, as reflected by the increased distance traveled at high speed, whereas these abnormal seizure-like behavior phenotypes were abolished under the treatment of TpNPY (0.75–6 μM). In a parallel experiment, we also examined the potential of TpNPY to reverse the alterations in seizure-related genes induced by PTZ in zebrafish. Specifically, we investigated the expression patterns of the immediate-early genes, including *c-fos* and *npas4a*, which are recognized to be upregulated upon PTZ treatment, as indicators of neuronal activation (Baxendale et al. 2012). As shown in Fig. 2D and E, the mRNA expression levels of these two genes were significantly upregulated in response to PTZ treatment. However, treatment with TpNPY successfully reversed this overexpression. Oxidative stress, ignited by excessive free radical release, is believed to be involved in both the initiation and progression of epilepsy (Shin et al. 2011). Low levels of ROS are crucial for the maintenance of normal cellular function. Consequently, prolonged elevation of ROS poses a significant risk of exacerbating neurodegeneration, as observed in conditions like epilepsy (Lin et al. 2020). The results presented in Fig. 2C and F demonstrated that the ROS levels in zebrafish larvae upregulated dramatically following PTZ challenge. Nevertheless, treatment with TpNPY mitigated the overaccumulation of ROS elicited by PTZ effectively.

### TpNPY suppressed seizure-like behavior, reduced the levels of inflammatory cytokines and ameliorated abnormal glial activation in the PTZ kindling epileptic model in mice

**Fig. 3:**
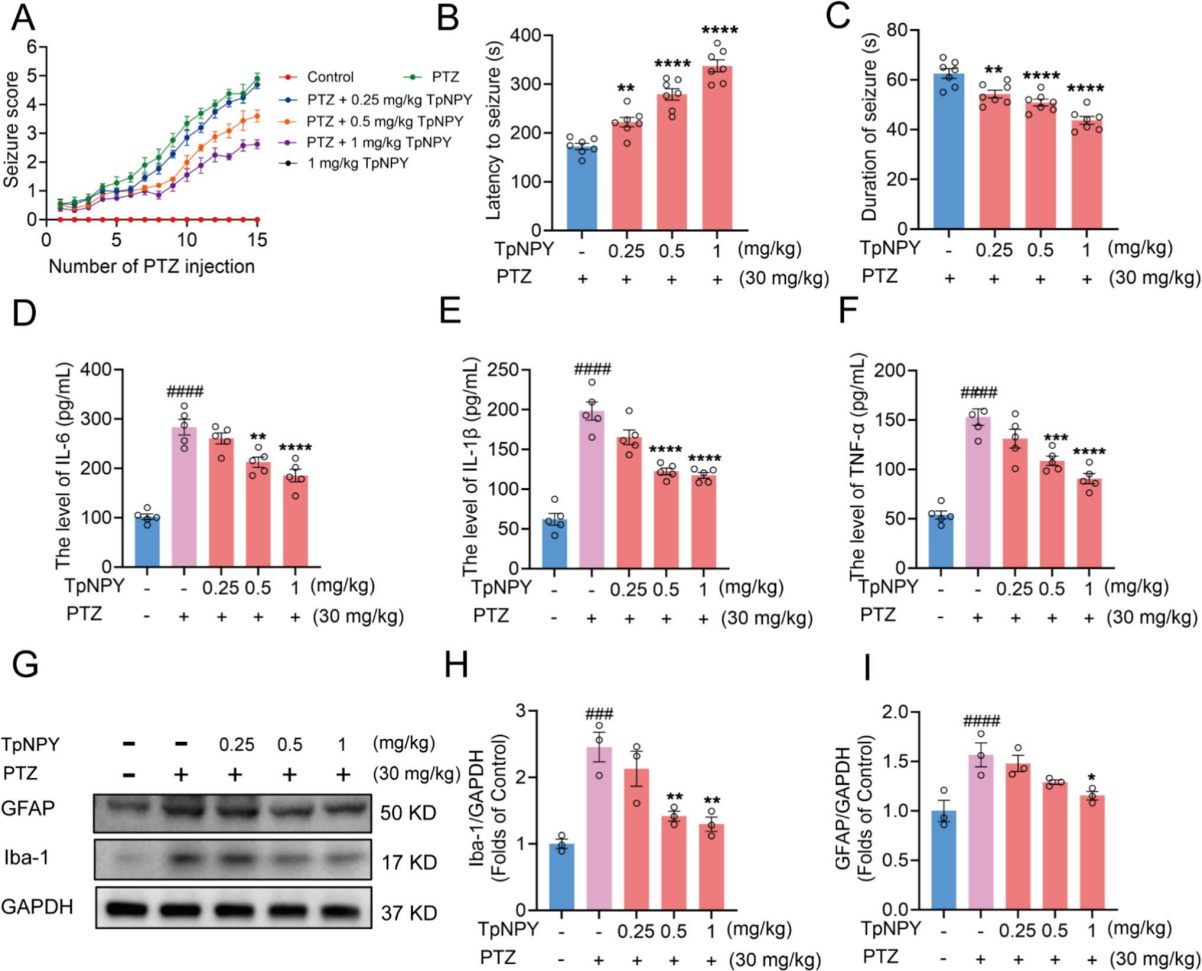
TpNPY suppressed seizure-like behavior, decreased inflammatory cytokines levels and ameliorated abnormal glial activation in PTZ kindling epileptic model in mice. **A** The seizure score of different treatment groups in mice. **B** The latency to generalized seizures of different treatment groups in mice. **C** The duration of generalized seizures of different treatment groups in mice. The inflammatory cytokine expression levels, including IL-6 **D**, IL-1β **E** and TNF-α **F**, in the brains of mice in different treatment groups. **G, H, I** Representative Western blot images of protein expression and densitometric quantification of protein expression of Iba-1 and GFAP. Data are expressed as Means ± SEM (*n* ≥ 3). ^###^*P* < 0.001, ^####^*P* < 0.0001 vs control, ^*^P < 0.05, ^**^P < 0.01, ^***^P < 0.001, ^****^P < 0.0001 vs PTZ group

We further explored the anticonvulsant potential of TpNPY in an in vivo PTZ kindling epilepsy model in mice. Phenotypic parameters, including seizure score, the latency of seizure (the time from administering PTZ to the first occurrence of seizure behavior), as well as the duration of seizure episodes, were evaluated. Mice receiving the same volume of saline were set as the control group. As indicated in Fig. 3A, after 15 injections, the seizure score in PTZ-treated group arrived at 4.91 ± 0.41; whereas TpNPY treatment at 0.25, 0.5, and 1 mg/kg reduced the seizure score to 4.69 ± 0.32, 3.60 ± 0.47, and 2.62 ± 0.35, respectively. Notably, TpNPY alone had no obvious influence on the behavior of mice. In addition, TpNPY dose-dependently augmented the latency to seizure generation, as well as decreased the duration of generalized seizures, implying that TpNPY exhibited anticonvulsant activity in PTZ kindling mice (Fig. 3B and C). Moreover, PTZ administration significantly upregulated the expression levels of IL-6, IL-1β, and TNF-α in the mouse hippocampus after 14 consecutive days of induction with a subconvulsive dose of PTZ (30 mg/kg). In contrast, TpNPY treatment effectively reversed these PTZ-induced elevations in proinflammatory cytokine expression (Fig. 3D-F). Glial fibrillary acidic protein (GFAP), a hallmark reflecting astroglial activation, has been shown to be upregulated in the brains of PTZ kindling epileptic mice (Pekny and Pekna 2014). Ionized calcium-binding adapter molecule 1 (Iba-1) functions as a molecular marker for identifying activated microglia in various neurological disorders (Norden et al. 2016). Abnormal microglial activation is a stimulus in the development of epilepsy (Wu et al. 2023). Likewise, the protein expression of GFAP and Iba-1 in the ventral hippocampus of mice was significantly increased upon subconvulsive dose of PTZ (30 mg/kg) challenge for consecutive 14-day; however, these upregulation trends were counteracted by the application of TpNPY, implying that it could exert inhibitory effects on PTZ-induced astrocytic and microglial activation (Fig. 3G-I).

### TpNPY attenuates PTZ-induced anxiety-like behaviors and promotes the impaired learning and spatial memory abilities in mice

**Fig. 4:**
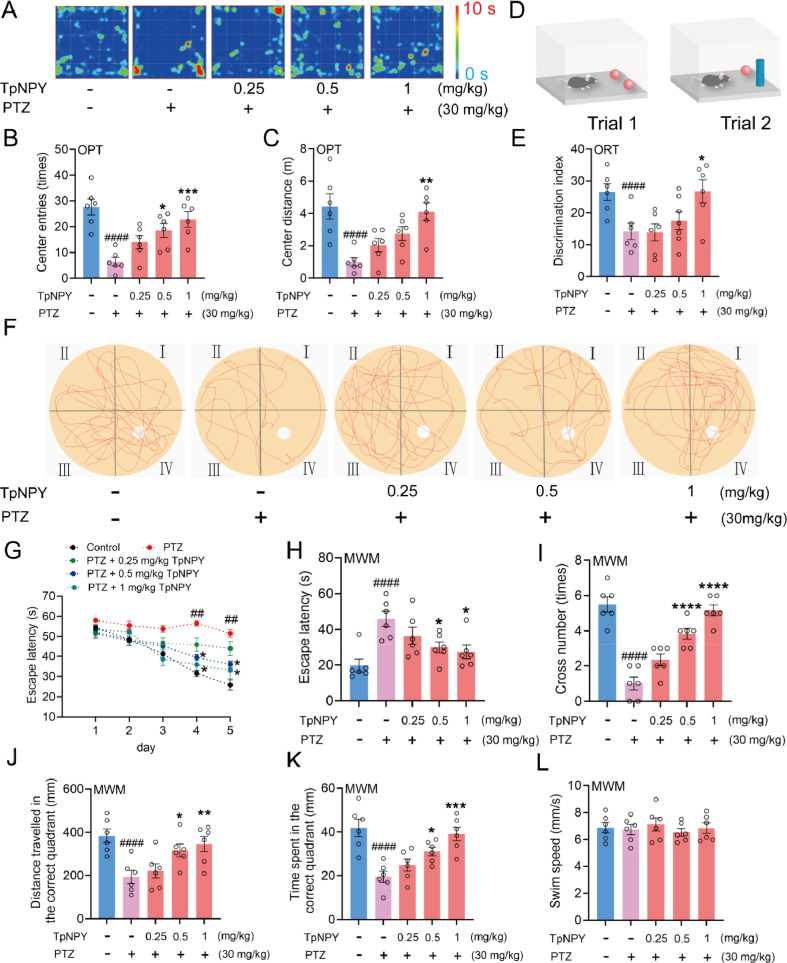
Effects of TpNPY on PTZ-induced anxiety-like behaviors, learning and spatial memory defects in mice. **A** The representative trajectory heatmaps of different treatment groups in mice in OFT. **B** Center entries and **C** center distance travelled of different treatment groups in mice in OFT. **D** Simplified schematic of the ORT experiment. **E** The discrimination index of different treatment groups in mice in ORT. **F** The representative movement trajectories of mice during the test stage of MWM. **G** The escape latency of mice spent during the training stage of MWM test. **H–L** the escape latency **H**; number of platform crossings **I**; distance traveled in the correct quadrant **J**; time spent in the correct quadrant **K**; swimming speed **L** during the test stage of MWM. Data are expressed as Means ± SEM (*n* = 6). ^####^*P* < 0.0001 vs control, ^*^P < 0.05, ^**^P < 0.01, ^***^P < 0.001, ^***^P < 0.0001 vs PTZ group

As illustrated in Fig. 4A-C, the open field test revealed that PTZ-treated mice displayed significantly increased anxiety-like behaviors, evidenced by a marked reduction in both time spent and distance traveled in the central zone compared to the control group. Notably, TpNPY administration substantially reversed these behavioral deficits, suggesting its efficacy in mitigating PTZ-induced anxiety. In the novel object recognition (NOR) test, PTZ-treated mice exhibited a significantly lower discrimination index for novel objects compared to the control group. However, TpNPY treatment restored this cognitive deficit, demonstrating its protective effect on object memory retention (Fig. 4D and E). Furthermore, in the Morris water maze (MWM) test (Fig. 4F), PTZ-treated mice showed prolonged escape latencies and fewer platform crossings compared to the control group (Fig. 4G-I). Additionally, PTZ-treated mice spent less time in the target quadrant and traveled shorter distances within it (Fig. 4J and K). Importantly, TpNPY treatment ameliorated these spatial memory impairments without affecting swimming speed, as no significant intergroup differences in locomotor performance were observed (Fig. 4L).

### The effects of TpNPY on neurogenesis, neural synaptic plasticity and BDNF/TrkB signaling pathway

**Fig. 5:**
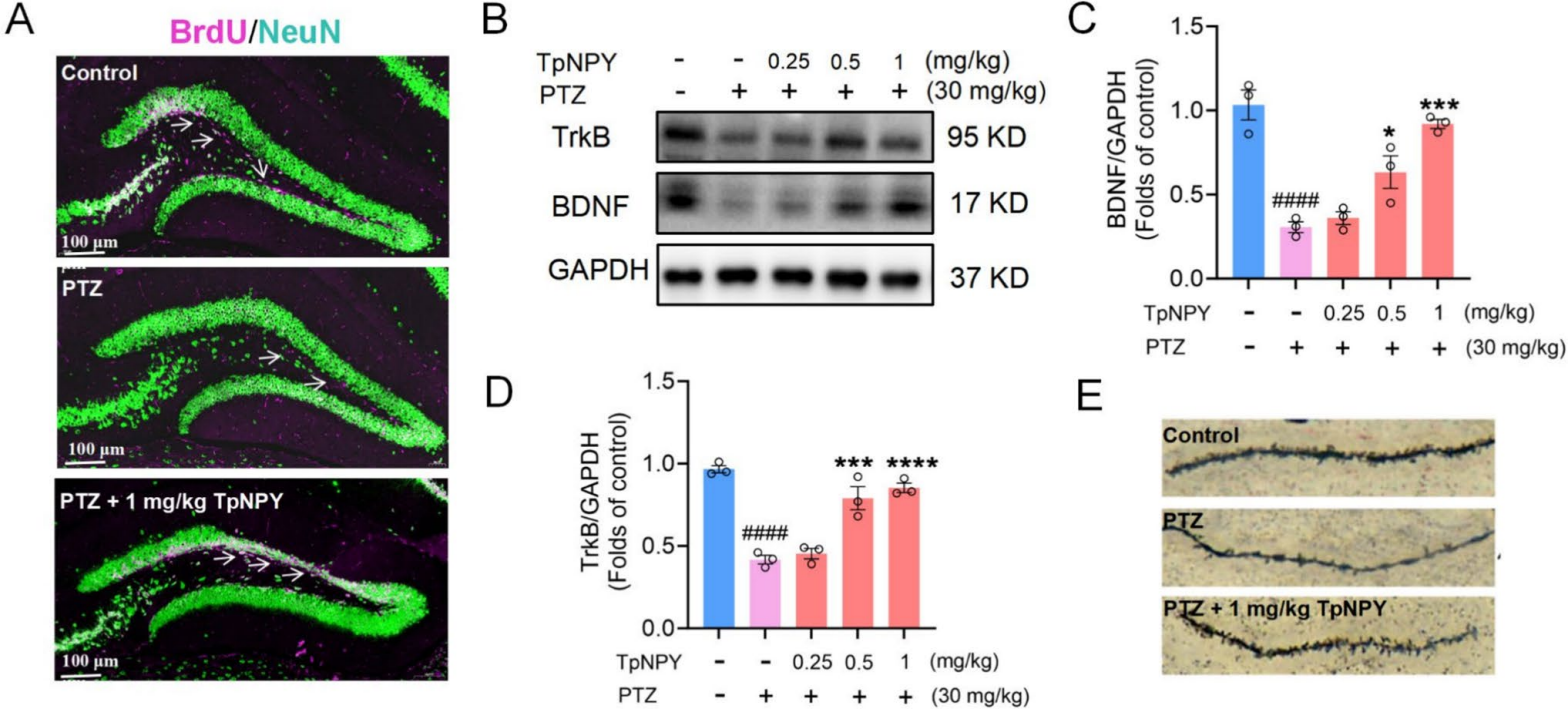
TpNPY promotes neurogenesis and neural synaptic plasticity through the BDNF/TrkB signaling pathway. **A** Neurogenesis in mice hippocampal DG with different treatment after chronic PTZ induction. Fluorescence confocal analysis of cells double-labeled for BrdU (pink) and NeuN (green). **B-D** The effects of TpNPY on the levels of TrkB and BDNF in the brains of mice challenged by PTZ. **E** Golgi-Cox staining of dendritic spines in the CA3 area in mice with different treatments. Data are expressed as Means ± SEM (*n* ≥ 3). ^####^*P* < 0.0001 vs control, ^*^P < 0.05, ^***^P < 0.001, ^***^P < 0.0001 vs PTZ group

Neurogenesis is reported to be involved in learning and spatial memory (Saxe et al. 2006). BrdU (bromodeoxyuridine), a thymidine analogue, is incorporated into the DNA of proliferating cells and serves as a marker for newborn neurons (Taupin 2007). As shown in Fig. 5A, chronic exposure to PTZ (14 days) suppressed the neurogenesis in the hippocampal dentate gyrus (DG) in mice, as reflected by decreased number of BrdU^+^ newborn neuronal cells, whereas the application of TpNPY significantly promotes the neurogenesis. Activation of the TrkB/BDNF signaling pathway potently promotes neurogenesis through facilitating neural stem cell proliferation, differentiation, neuronal survival, and functional synaptic integration (Li et al. 2008). As demonstrated in Fig. 5B-D, PTZ treatment significantly decreased the expression levels of TrkB and BDNF, while the administration of TpNPY dose-dependently upregulates these two proteins. Moreover, TpNPY promotes neural synaptic plasticity as depicted in Fig. 5E. Collectively, these findings demonstrate that TpNPY promotes neurogenesis and neural synaptic plasticity through the BDNF/TrkB signaling pathway.

### TpNPY restored cell damage in glutamate-elicited HT22 cells

**Fig. 6:**
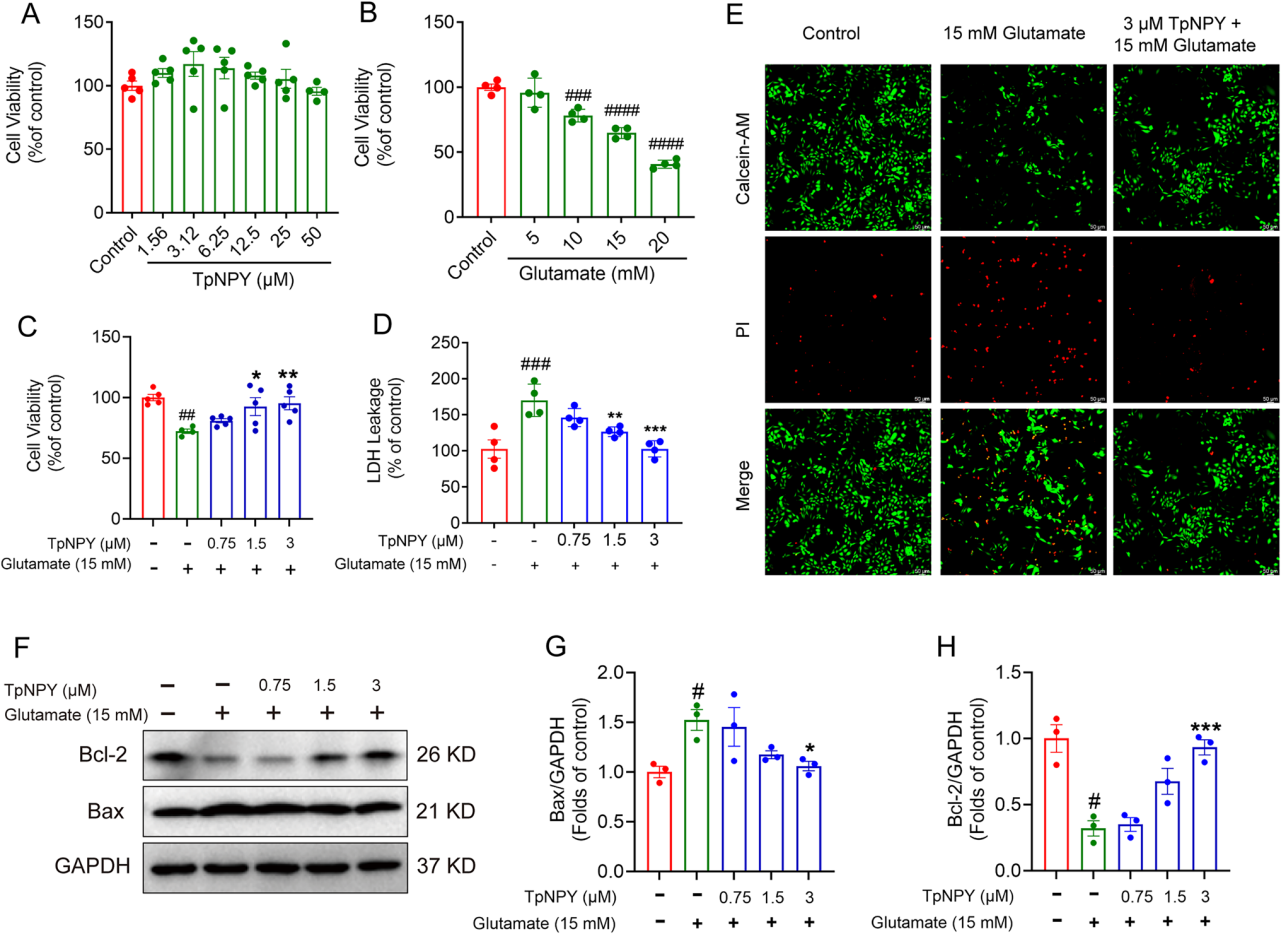
TpNPY restored HT22 cell damage challenged with glutamate. **A** HT22 cell viability after 24 h exposure to various concentrations of TpNPY. **B** HT22 cell viability after 24 h incubation with different concentrations of glutamate. **C** HT22 cell viability following 24 h incubation with increasing concentrations of TpNPY with or without 15 mM glutamate. **D** LDH leakage of HT22 cells after incubation with indicated concentrations of TpNPY in the presence or absence of 15 mM glutamate for 24 h. **E** PI/Annexin V staining of HT22 cells in different treatment groups. **F–H** Representative Western blot images of the expression of Bcl-2 and Bax, as well as the quantitative data of their expression levels in HT22 cells with different treatments. Data are shown as Means ± SEM (n ≥ 3). ^#^*P* < 0.05, ^##^*P* < 0.01, ^###^*P* < 0.001, ^####^*P* < 0.0001 versus control group. ^*^P < 0.05, ^***^P < 0. 01, ^***^P < 0.001 versus glutamate-challenged group

The immortalized mouse hippocampal cell line, HT22 cells, which are characterized by lacking functional ionotropic glutamate receptors, are known for their susceptibility to excitotoxicity (Kritis et al. 2015). HT22 cells have been regarded as an efficient in vitro cell model for investigating the interplay between glutamate-induced calcium ion influx and oxidative damage in neuronal cells (Tan et al. 1998). In order to evaluate the protective potential of TpNPY against glutamate-induced neuronal toxicity, we first examined the cytotoxicity of TpNPY on HT22 cells. The results demonstrated that TpNPY ranging from 1.56 to 50 μM did not elicit any significant changes in cell viability after 24 h incubation (Fig. 6A). To verify the toxic effects of glutamate on HT22 cells, various concentrations of glutamate (ranging from 5 to 20 mM) were employed for a 24 h incubation period. It was observed that challenged by increasing concentrations of glutamate led to a dose-dependent decrease in cell viability. Specifically, treatment with 10 mM, 15 mM, and 20 mM glutamate reduced cell viability to 78.22%, 64.78%, and 40.82%, respectively (Fig. 6B). Thus, a 24 h intervention with 15 mM glutamate was chosen to cause cellular injury in HT22 cells for subsequent experiments. Notably, the reduced cell viability triggered by glutamate was restored by the incubation of TpNPY, as verified by the MTT assay, LDH assay and live-dead cell staining assay (Fig. 6C-E). Bax (Bcl-2-associated X protein) is a pro-apoptotic protein, which is essential for maintaining tissue homeostasis and eliminating damaged cells. Bax can be regulated by other members of the Bcl-2 family, including anti-apoptotic proteins like Bcl-2 itself (Cory et al. 2003). During apoptosis, various signals or stressors can trigger the activation of Bax. As shown in Fig. 6F-H, glutamate stimulation significantly decreased the expression of Bcl-2 and increased the expression of Bax in HT22 cells, while treatment with TpNPY abolished these effects, suggesting that TpNPY decreases the apoptosis of glutamate-injured HT22 cells in a dose-dependent manner.

### TpNPY attenuated oxidative stress indicators and intracellular calcium ion overaccumulation of HT22 cells challenged with glutamate via Nrf2/HO-1 signaling pathway

**Fig. 7:**
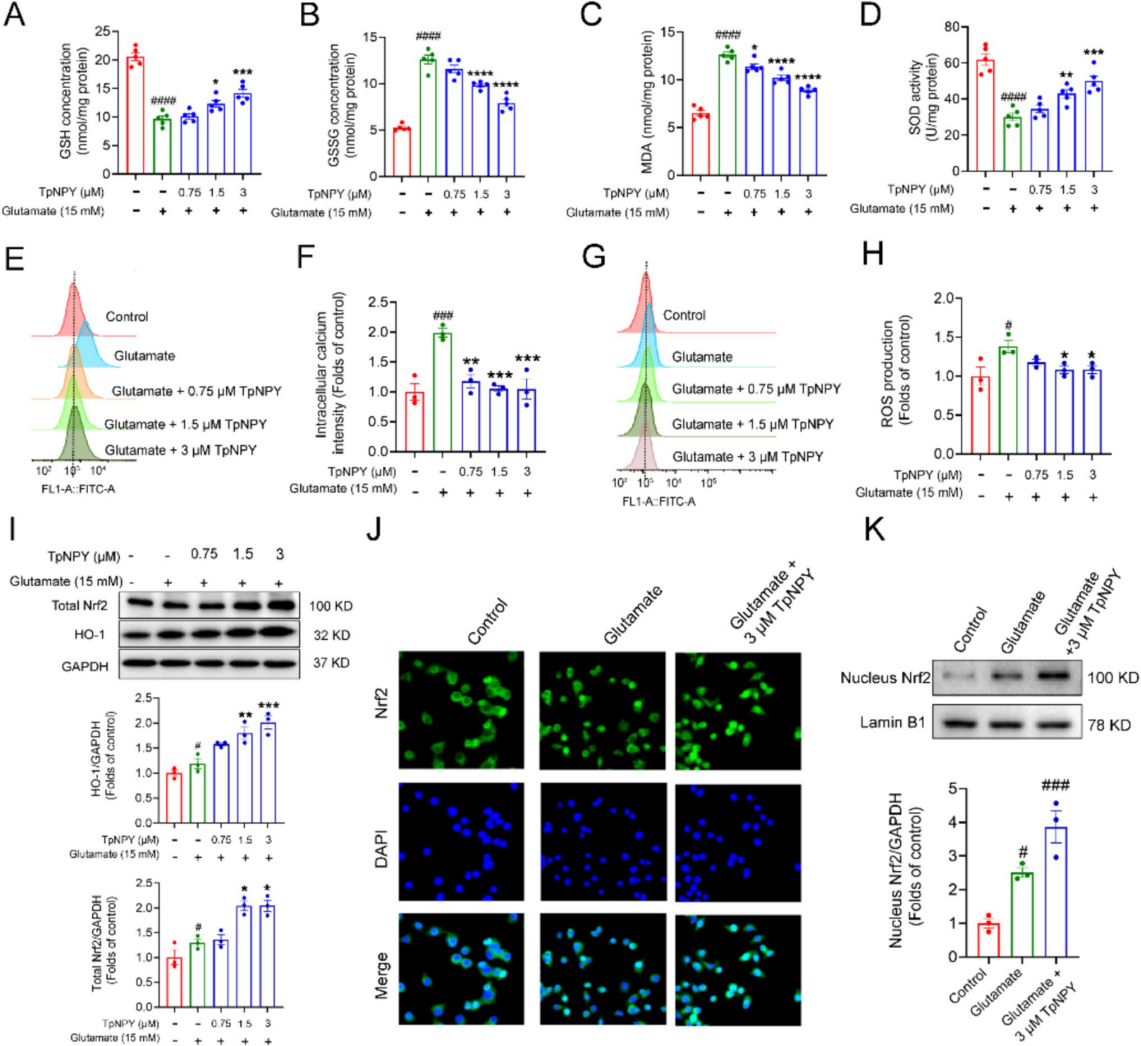
TpNPY attenuated oxidative stress indicators and intracellular calcium ion overaccumulation of HT22 cells challenged with glutamate through Nrf2/HO-1 signaling pathway. The concentration of **A-C** GSH, GSSG, MDA and **D** SOD activity in HT22 cells. **E–H** TpNPY reduced the intracellular calcium ion accumulation **E** and **F** and ROS generation **G** and **H** in glutamate-challenged HT22 cells, the cells were cotreated with 15 mM glutamate and in the presence or absence of increasing concentrations of TpNPY for 12 h. **I** Representative Western blot images showing the effects of TpNPY on Nrf2/HO-1 expression levels and quantitative data of their expression levels of HT22 cells after challenged with glutamate. **J** The Nrf2 protein localization was detected by immunofluorescence staining. The Nrf2 proteins were labeled via Nrf2 primary antibody and Alexa Fluor 488-conjugated secondary antibody. The nucleus was counterstained with DAPI. **K** Representative Western blot images of Nucleus-Nrf2 and quantitative data in each group. Data are presented as Means ± SEM (n ≥ 3). ^#^*P* < 0.05, ^###^*P* < 0.001, ^####^*P* < 0.0001 versus control group. ^*^P < 0.05, ^**^P < 0.01, ^***^P < 0.001, ^****^P < 0.0001 versus glutamate-treated group

Furthermore, the activity of SOD, the expression levels of MDA, GSH and GSSG, in each treatment group were investigated to explore whether TpNPY alleviated the oxidative stress elicited by glutamate in HT22 cells. SOD activity, MDA and GSH concentration in the glutamate-treated group reduced sharply and GSH concentration increased dramatically, whereas these effects were effectively counteracted by the application of increasing concentration of TpNPY (Fig. 7A-D). These findings demonstrated that TpNPY effectively reversed the alterations in oxidative indicators caused by glutamate, thereby restoring cellular oxidative levels to normal condition. Moreover, the application of TpNPY dramatically prevented glutamate-induced calcium overload (Fig. 7E and F) and ROS accumulation (Fig. 7G and H). To evacuate the potential mechanism of the protective effects of TpNPY on glutamate-challenged HT22 cells, the HO-1 and Nrf2 expression levels were evaluated. Notably, TpNPY dose-dependently upregulated the protein levels of Nrf2 and HO-1 compared to the glutamate-challenged group (Fig. 7I). The Nrf2 translocation from the cytoplasm to the nucleus plays a crucial role in its activation, leading to the subsequent upregulation of antioxidant and detoxification genes (Baird and Yamamoto 2020). Immunostaining analysis and Western blot results, as depicted in Fig. 7J and K, revealed a significant enhancement in the nuclear translocation of Nrf2 in HT22 cells following the application of TpNPY, in comparison to the other two groups. Collectively, these findings indicated that TpNPY conferred protection to HT22 cells against glutamate-induced toxicity by activating the Nrf2/HO-1 pathway, thereby to inhibit the increase of oxidative stress and intracellular calcium levels.

## Discussion

For thousands of years, corals have been utilized in a variety of traditional Chinese medicine formulas; medicinal corals can tranquilize the mind and display therapeutic effects in the treatment of epilepsy (Han et al. 2023). As medicinal corals are primarily administered as compound preparations, their active anti-epileptic components remain incompletely characterized. Previously, our team identified several Kunitz-type polypeptides from corals that exhibit anti-epileptic effects through the interaction with the gamma-aminobutyric acid A (GABA_A_) receptor and the transient receptor potential vanilloid 1 (TRPV1) channel (Chen et al. 2022a; Wang et al. 2021). These findings suggest that these understudied coral-derived polypeptides harbor tremendous potential in the development of anti-epileptic agents.

Neuropeptides are crucial signaling molecules that facilitate communication between neurons and glial cells (Carniglia et al. 2017). Their functions extend beyond simple neurotransmission, influencing various neurophysiological processes. Research continues to uncover the complexities of neuropeptide interactions. The multifaceted roles of neuropeptides in both healthy and diseased states underscore the importance of studying these molecules. Various neuropeptides, including substance P, neuropeptide Y, and endorphins, participate in diverse pathways that regulate functions such as appetite, mood, and circadian rhythms (Hökfelt et al. 2003). Recent studies highlight the therapeutic potential of NPY signaling modulation in epilepsy. Endogenous neuropeptide Y has been proved to play a regulatory role in epilepsy by modulating neuronal excitability (Vezzani et al. 1999). Mice lacking the NPY gene display exacerbated susceptibility to seizures (Erickson et al. 1996), whereas rats overexpressing the NPY gene exhibit decreased seizure susceptibility and inhibit epileptogenesis (Vezzani et al. 2002). Furthermore, exogenous administration of NPY can alleviate picrotoxin-induced seizure-like behavioral phenotypes in rats (Smiałowska et al. 1996). Considering that TpNPY exhibits sequence similarity to mammalian NPY and directly interacts with NPY-Y1R, this study reveals its significant efficacy in treating seizures, demonstrating the potential for discovering and developing novel drug candidates from neuropeptides derived from marine organisms.

Neural hyperactivity, usually accompanied by excitotoxicity and manifested by immediate early response genes, is regarded as a cause of convulsion (Pan et al. 2018). Pentylenetetrazole is a classical GABA receptor antagonist, which increases neural activity by restraining the function of inhibitory neurotransmitters, thereby leading to seizures (Nieoczym et al. 2024). Seizure-like behavior in zebrafish larvae can be reliably induced by directly immersing them in PTZ solution, which facilitates high-throughput drug screening with anti-epileptic or anti-seizure efficacy (Baraban et al. 2005). In the present study, we observed that PTZ treatment triggered abnormal swimming patterns and IER gene (*c-fos* and *npas4a*) overexpression in zebrafish larvae. As molecular biomarkers related to neuronal activities, *c-fos* and *npas4a* have been previously confirmed to be upregulated during seizure events (Baraban et al. 2005). *c-fos* serves as the gold standard for assessing synaptic function in the central nervous system (CNS) and can be transiently induced in response to the onset of seizures (Torres-Hernández et al. 2016). The transcription factor *npas4a* regulates the development of inhibitory synapses by controlling the expression of a number of activity-dependent genes, thereby maintaining the equilibrium between excitatory and inhibitory synapses within neural circuits (Lin et al. 2008). Interestingly, TpNPY dramatically attenuated the PTZ-induced epilepsy-like behavior in zebrafish larvae, as reflected by the total distance travelled at high speed. Consistent with the locomotion test results, the PTZ-stimulated overexpression of *c-fos* and *npas4a* was dramatically reduced by TpNPY treatment, suggesting that TpNPY inhibited IER gene expression and thereby regulates the homeostasis between synaptic excitatory and inhibitory activities.

Neuroinflammation is a pivotal mechanism involved in the pathophysiology of epilepsy, and it is regarded as a biomarker indicating the process of epileptogenesis (Vezzani and Friedman 2011). Inflammatory mediators can lead to neuronal hyperexcitability through the activation of specific signaling pathways, thereby increasing the possibility of seizure recurrence. Epilepsy, in turn, triggers overactivation of astrocytes and the inflammatory process, aggravating brain damage (Nonato et al. 2024). Notably, anti-inflammatory treatment effectively attenuated the frequency of spontaneous seizures and mitigated the severity of this disease (Vezzani 2015). Our recent study revealed that TpNPY exhibits anti-neuroinflammatory activity in C8-D1A astrocytes in vitro (Chen et al. 2022b), while its efficacy in vivo is still unknown. In this study, the experimental results indicated that TpNPY improved seizure behavior in the PTZ-kindled mouse model, as evidenced by the reduced seizure score, the shortened duration of seizure episodes, and the increased latency of seizure. Strikingly, these behavior phenotypes occurred concurrently with the downregulation of inflammatory cytokines and abnormal glial activation. The efficacy of TpNPY in vivo suggested its potential to pass through blood–brain barrier and affect the brain microenvironment and neurofunctions, which could permit its therapeutic development in the future.

Epileptic attacks can cause an increase in the incidence of anxiety, cognitive decline and memory deficits (Ammothumkandy et al. 2025; Javaid et al. 2024). The widely distributed NPY in the CNS regulates various physiological processes, including stress response, anxiety, and cognition (Borroto-Escuela et al. 2022). NPY1R is the most dominant NPY receptor (NPYR) subtype in the brain and is essential for mood regulation (Lach and de Lima 2013). Besides, NPY1R in the hippocampus is involved in memory and spatial learning (Borroto-Escuela et al. 2022). NPY1R agonist promotes hypothalamic neurogenesis in a rat experimental depression model (Solak et al. 2024). When exposed to stress stimuli, the wild-type rats exhibited an anxiogenic effect, which was absent in the NPY-overexpressing rats (Thorsell 2008). Moreover, the intracerebroventricular administration of NPY can mitigate both anxiety and depression-associated behaviors in diverse animal models, predominantly via its binding to NPY1R (Solak et al. 2024). The anxiolytic effect is comparable in potency to benzodiazepines in rats (Dahan et al. 2024). As for the mediation effects of NPY in the cognitive process, it is reported that the intracerebroventricular injection of NPY or NPY1R agonist improves spatial memory deficits in rats with an AD-like phenotype (Rangani et al. 2012). Neurogenesis is the process of generating new neurons in the brain, which is related to the spatial and learning memory (Niklison-Chirou et al. 2020). It is well-established that chronic seizures damage and exhaust adult neural stem cells, leading to a decline in adult neurogenesis within months of seizures post-induction (Toda et al. 2019). In the hippocampus of aged rats, the decreased NPY expression was associated with memory impairment and abnormal neurogenesis (Borbély et al. 2013). Likewise, both NPY receptor densities in hippocampus and cortical regions (Martel et al. 1990) and NPY levels in plasma and cerebrospinal fluid samples (Nilsson et al. 2001) were reduced in AD patients. The BDNF/TrkB signaling pathway critically regulates neurogenesis through pleiotropic mechanisms. BDNF binding to TrkB receptors activates PI3K/Akt and MAPK/ERK cascades, thereby promoting neural stem cell (NSC) proliferation in the subgranular zone (SGZ) of the hippocampus (Wang et al. 2024). Besides, BDNF/TrkB signaling inhibits pro-apoptotic BAD protein via Akt, enhancing the survival of newborn neurons (Liu and Song 2016). In this study, the administration of TpNPY significantly decreased the PTZ-induced anxiety-related behavior in mice and improved the recognition memory deficits, as confirmed by the results of neurobehavior tests of OFT, NOR and MWM. Strikingly, these effects were accompanied by the enhanced neurogenesis and improved synaptic plasticity in mice hippocampus treated with TpNPY, suggesting that TpNPY may prevent PTZ-induced anxiety-like behaviors and recognition memory deficits by promoting neurogenesis through the upregulation of BDNF/TrkB signaling pathway.

Apart from neuroinflammation, aberrant calcium flow and oxidative stress are considered to be the molecular basis of epileptogenesis, leading to seizure-triggered neuronal cell death, increased vulnerability to neuronal synchronization and network changes (Ambrogini et al. 2019). Enhanced glutamate release can be observed in epileptic patients’ brain tissues, as well as in animal models of seizures (Coulter and Eid 2012). Usually, the excitotoxicity induced by glutamate can lead to an excessive generation of reactive oxygen species (ROS), thereby initiating neuronal cell death through oxidative stress (Murphy et al. 1989). ROS production plays a crucial role in epileptogenesis, functioning as both a contributor to the development of epilepsy and a consequence of the condition (Patel 2004). Elevated intracellular Ca^2+^ concentration is also deemed as a characteristic of neuronal cell death resulting from glutamate-induced oxidative stress (Fukui et al. 2009). Interestingly, mammalian NPY can exert robust inhibitory effects on presynaptic glutamate release, effectively eliminating the extent of provoked excitatory responses in the hippocampus (Colmers et al. 1985). HO-1 is considered as a pivotal regulator in modulating the biological oxidative stress system, which possessed both in vivo and in vitro neuroprotective effects (Barone et al. 2014; Schipper et al. 2009). The expression of HO-1 protein is controlled by the upstream transcription factor Nrf2. Interestingly, the Nrf2-mediated signaling pathway triggers endogenous antioxidant responses to counteract oxidative stress and simultaneously facilitates the removal of ROS (Loboda et al. 2016). In the present study, our findings demonstrated that the manipulation of TpNPY was able to recover the glutamate-induced HT22 cell damage, as well as prevent the excessive aggregation of intracellular ROS and overproduction of calcium ion concentration (Fig. 5). These effects were accompanied by the restoration of antioxidant levels such as GSSG, GSH, SOD and MDA. In addition, TpNPY upregulated the expression levels of Nrf2 and HO-1 in glutamate-challenged HT22 cells and promoted the translocation of Nrf2, indicating that TpNPY rescued glutamate-induced cell damage through its anti-oxidative stress via Nrf2/HO-1 signaling pathway.

## Conclusions

In summary, our findings demonstrate that TpNPY exerts potent anti-epileptic activity in both zebrafish and murine PTZ-induced seizure models via specific interaction with NPY-Y1R. The observed therapeutic effects were primarily mediated through dual mechanisms of anti-inflammatory action and oxidative stress mitigation. Furthermore, TpNPY administration significantly enhanced hippocampal neurogenesis and synaptic plasticity via activation of the TrkB/BDNF signaling cascade, resulting in measurable improvements in cognitive function and spatial memory retention in treated animals. These results provide compelling preclinical evidence supporting the development of this marine-derived peptide as a promising therapeutic candidate for epilepsy and neuroinflammation-associated neurological disorders.

## Data Availability

The datasets generated during and/or analyzed during the current study are available from the corresponding author on reasonable request.
